# Microbicides 2006 conference

**DOI:** 10.1186/1742-6405-3-25

**Published:** 2006-10-13

**Authors:** Gita Ramjee, Robin Shattock, Sinead Delany, Ian McGowan, Neetha Morar, Megan Gottemoeller

**Affiliations:** 1 HIV Prevention Research Unit, Medical Research Council, Durban, South Africa; 2 Centre for Infection, Cellular and Molecular Medicine, St. George's University of London, London, UK; 3 Reproductive Health and HIV Research Unit, University of Witwatersrand, Johannesburg, South Africa; 4 UCLA AIDS Institute, University of California, Los Angeles, USA; 5 Global Campaign for Microbicides, Washington, DC, USA

## Abstract

Current HIV/AIDS statistics show that women account for almost 60% of HIV infections in Sub-Saharan Africa. HIV prevention tools such as male and female condoms, abstinence and monogamy are not always feasible options for women due to various socio-economic and cultural factors. Microbicides are products designed to be inserted in the vagina or rectum prior to sex to prevent HIV acquisition.

The biannual Microbicides conference took place in Cape Town, South Africa from 23–26 April 2006. The conference was held for the first time on the African continent, the region worst affected by the HIV/AIDS pandemic. The conference brought together a record number of 1,300 scientists, researchers, policy makers, healthcare workers, communities and advocates. The conference provided an opportunity for an update on microbicide research and development as well as discussions around key issues such as ethics, acceptability, access and community involvement.

This report discusses the current status of microbicide research and development, encompassing basic and clinical science, social and behavioural science, and community mobilisation and advocacy activities.

## Background

It has been twenty five years since HIV/AIDS was first recognized, with disproportionate number of infections among women. Sub-Saharan Africa houses the largest number of HIV infected women. It is thus not surprising that women are now the focus of HIV prevention efforts. Microbicides are products designed to be women initiated which could have the potential to prevent HIV infection, should she not be able to negotiate condom use with her partner. The products can be formulated as gels, creams and suppositories, and could be inserted in the vagina prior to sex. It is hoped that these products would have a bidirectional effect; prevent male to female and female to male transmission of HIV. In addition, the products are also being tested for safety during rectal use.

Recently, microbicides have attracted a lot of attention in the media, with commitments for accelerated research coming from philanthropic and public sectors. The biannual Microbicides conference was held earlier this year in Cape Town, South Africa. The conference attended by more than 1,300 delegates was held for the first time on the African continent. In this issue, we report on some of the key messages emanating from this very important conference. The conference agenda covered recent developments in basic, clinical and social science, and for the first time in the history of the conference, included a community track. This track covered community partnerships and involvement, donor panels and general discussions on microbicide advocacy.

The outcomes of each track are summarised below.

## Track A: Basic Science

### Robin Shattock

The basic Science track (Track A) reviewed the current status of microbicide design and development. In her plenary presentation, Prof Sharon Hillier (University of Pittsburg, USA) demonstrated the microbicide discovery pipeline which has a broad diversity of compounds acting at different stages in the viral lifecycle, each exhibiting significant strengths and challenges for development. In reviewing different classes of product such as surfactants, acid buffers, polyanians, etc, we are seeing an increasing push for drugs with higher potency. First generation products (including membrane disrupting compounds, non-specific fusion inhibitors and acid buffering agents), while having broad activity against a range of sexually transmitted diseases, are limited by the requirement for coitally dependent use with moderate or lower potency against HIV. Recent research has focussed on the push for drugs with increasing potency which has only been achievable through the targeting of specific components of the viral life cycle (attachment, fusion and reverse transcription), often at the expense of activity against other sexually transmitted diseases. This means that products with high anti-HIV potency are not likely to act against sexually transmitted pathogens such as *Chlamydia *and *Gonorrhoea*. The richest pipeline of new compounds entering clinical development is the use of antiretroviral drugs (ARVs) in new formulations. These drugs have high specificity and potency. The use of ARVs could for the first time give rise to the development of some degree of coitally independent microbicides delivered either as a daily application, or through sustained delivery devises such as intravaginal rings currently used for contraception. The potential downside of such products is the lack of efficacy against other sexually transmitted infections (STIs) and the potential for resistance evolution.

A plenary by Professor Mark Wainberg's (AIDS Centre, Montreal, Canada) discussed the issues and implications surrounding the use of ARVs in microbicides. While resistance to individual ARV-containing microbicides is possible if used by women unaware of their HIV positive status, the limited systemic absorption of such products would reduce likelihood of such evolution. However, a more pressing concern regarding resistance, could be the transmission of the virus that is resistant to the compounds contained in the microbicide formulation. Nevertheless, it is likely that the use of combination ARVs would be the next logical step to prevent resistance evolution, analogous to current treatment regimes. Given some of the challenges, the development of an effective product is likely to be a long process. Ultimately an effective microbicide would most likely be a combination product, providing protection against HIV-1 and other STDs and possibly combined with different barriers such as vaginal diaphragms for example, or delivery technologies such as vaginal rings – a true hybrid product.

One of the most important corner stones of microbicide development in addition to efficacy is safety and certainly no microbicide candidate should disrupt natural barriers to infection. Work presented in Track A demonstrated significant progress in identifying potential biomarkers of safety in preclinical cellular, tissue and animal models. Betsy Harold (Mount Sinai Hospital, New York, USA) presented a novel murine model using sub-lethal challenge with HSV infection that could provide a functional assay to assess candidate safety [1]. Riana Fichorova (Harvard Medical School, USA) and Gustavo Doncel (CONRAD Eastern Virginia Medical School, USA) presented data on a range of inflammatory cytokines and epithelial expression of Cyclooxygenase, as markers of vaginal inflammation that could provide a matrix of parameters to predict in vivo safety [2,3]. The delicate nature of innate barriers to infection was elegantly demonstrated by Cecilia Cheng-Mayer (Aaron Diamond AIDS Research Center, USA) studying the effects of depoprovera on susceptibility of rhesus macaques to diverse viral strains. Depoprovera, which reduces the natural barrier provided by cervicovaginal epithelium, rendered animals more susceptible to a wider diversity of viral strains and lead to dominance of HIV-1 X4 viral replication [4].

Leonid Margolis (NICHD, Bethesda, USA) discussed how the selective transmission of HIV-1 R5 viruses suggested that there are a number of natural gatekeepers which selectively block mucosal transmission of HIV-1 X4 strains. These multiple gatekeepers pointed to the importance of blocking HIV-1 transmission at more than one level, providing a strong rationale for a combination approach to effective microbicide development as mentioned by Professors Hillier and Wainberg. Giving examples of natural pathogens that negatively impact on HIV replication (including Measles, HHV6 and 7), Margolis suggested they could unlock potential strategies for inducing protective innate responses [5].

This theme was taken up by Dr Maddy Hayes (St George's University of London, UK) who demonstrated that innate resistance to HIV infection could be induced in mucosal explants by immuno-modulatory oligonucleotides. The future focus of this work is to define the mechanisms of innate resistance and find ways to boost this in vivo. However, defining the mechanism of innate resistance to HIV in humans and its modulation by microbicides is perhaps someway from being realized. The current products have been actively evaluated for resistance evolution in cellular and animal models [6].

One of the more promising approaches is the targeting of the cellular co-receptor CCR5 utilized by the majority of transmitted strains. Two candidates in this category demonstrated high levels of protection in macaque challenge models [7,8]. Furthermore, work presented by John Moore demonstrated that combination of entry inhibitors could further enhance the levels of protection provided by blockade or CCR5 alone [7]. Drs Donald Mosier and Eric Arts (Case Western Reserve University) suggested that PSC-RANTES had a high barrier for resistance, most likely related to its ability to cause sequestration of the inhibitor within target cells [9]. In contrast work by Dr Qinxue Hu (St George's University of London, UK) demonstrated that resistance to Cyanovirin, that targets the virus through binding to gp120, could evolve with only three mutations, but the in vivo significance was unclear [10]. Furthermore, the activity of a wide range of antiviral plant lectins (including cyanovirin), presented by Dr Yven Van Herrewegewith (Institute of tropical Medicine, Belgium), may allow the development of combinations that further restrict the potential for resistance evolution [11].

The Basic science track also covered the issue of microbicide formulation leading to the development of microbicide products appropriate to wide-scale use. Professor David Woolfson (Queens University Belfast) presented the current state of the art for microbicide formulations and drug delivery. Appropriate formulation is seen to be essential to make a usable product in terms of efficacy and acceptability. Microbicides can be delivered through a variety of drug delivery devises, gel, creams, tablets, pessaries and controlled release devises such as intravaginal rings. Each technology has different strength and weakness. While current trials are focussed on the use of gels applied with every act of intercourse, newer technologies are being pursued that will allow products to be applied on a daily of weekly basis. Perhaps the most promising new technology is the development of microbicide release from intravaginal ring (IVRs), already used for hormonal contraception. Recent data from David Woolfson's group suggests that these rings could release preventive levels of antiretroviral drugs (ARVs) for greater than six months. While such technology is best adapted to delivery of small hydrophobic drugs, recent modifications suggest that they could be adapted to release a wider range of compounds, including peptide or protein based microbicides. A number of additional approaches are under development, including novel vaginal delivery systems which are triggered on contact with semen allowing drug release only following ejaculation (Patrick Kisser, University of Utah, USA) [12].

The Basic Science track demonstrated that there are a wide range of microbicide candidates at different stages of development, providing a broad width of strategies to prevent mucosal HIV transmission. The future development of the field is likely to focus on development of agents with increasing potency and the use of sustained delivery technology to provide prolonged protection.

## Track B: Clinical Science

### Sinead Delany-Moretlwe

The clinical track reviewed microbicides and other technologies such as the vaginal diaphragm in the prevention of HIV. Results of safety studies together with an update of current Phase III trials were presented.

### Phase I/II Safety trials

A long term safety study of praneem (a polyherbal vaginal tablet), when compared with placebo gel, was shown to be safe and acceptable in a study conducted in 100 HIV negative, monogamous women. 56% of women experienced an adverse event, but the majority of these (79%) were considered mild. Only 0.7% experienced severe adverse events. Most of the adverse events were associated with genital discomfort. Other adverse events observed included nausea associated with the smell of the product, intermenstrual bleeding and dysuria. There was no difference in the frequency or severity of adverse events observed between women in the praneem and placebo groups. Given the safety profile of praneem, it was recommended that this product be advanced to phase III trials [13].

A 14-day randomized, controlled safety and acceptability trial of Acidform™ compared with KY Jelly demonstrated that Acidform™ is more likely to cause genital irritation than KY Jelly (OR 2.6, p = 0.009). Although more peeling was observed on colposcopy in the Acidform™ group (25% vs. 4%), there appeared to be a decrease in pro-inflammatory cytokines observed in Acidform users. The study confirmed Acidform's potential to maintain the acidic environment of the vagina, with statistically significant differences in pH observed between the two groups. However, the changes to the vaginal flora were minimal. A greater proportion of women in the Acidform™ group found the gel unpleasant or neutral [14].

A randomized controlled trial of 0.5% PRO 2000 demonstrated that compared with placebo, this product is non-inflammatory after 14 days of application in a group of low risk women. A similar rate of adverse events was observed in both groups (70%). The most frequent symptom was genital burning. No increase in pro-inflammatory cytokines was observed in the PRO 2000 group. A trend toward increased anti-HSV activity was observed at day 7, but the clinical significance of this could not be determined at this stage [15].

A 3-arm phase I safety study examined the effect Carraguard compared with placebo or no gel, on cell-free HIV in the genital secretions of 40 HIV infected women. The results showed that Carraguard does not influence HIV shedding in the genital tract; no change in levels of HIV was observed in this group. As expected, levels of HIV in the genital tract correlated strongly with levels of HIV in the plasma [16].

A pre-phase I study to characterize the rectal mucosal safety parameters showed that studies involving regular sigmoidscopy and biopsy are feasible to conduct; and that cytokine and T cell measurements are useful markers of safety. These markers were not influenced by sexual practice. This suggests that it will be possible to identify meaningful endpoints for Phase 1 RM safety studies [17].

Two studies examining the safety of lime juice as a potential microbicide concluded that lime Juice did not appear to be safe in concentrations of ≥ 50%, the concentration required to inactivate HIV in semen [18].

### Barrier methods

Several trials demonstrated that physical barriers when used in combinations with gels were safe, acceptable and in some cases effective as contraceptives.

A 14-day randomized, controlled trial examined the safety of a silicone diaphragm when used in combination with either Buffergel, Acidform or KY Jelly (as placebo) in 81 low risk women. Adverse events were generally mild. 45 women reported experiencing adverse events; 26 of which were possibly related to study product. However, no serious adverse events were observed. The most frequent adverse events reported included backache, and genital discomfort. Severe findings on colposcopy were observed in two participants, both of whom were in the Acidform group. Colposcopic findings were generally more frequent in the two gel arms. The authors recommended that both these combinations be evaluated in Phase III trials [19].

Another randomized controlled trial evaluated the safety of the Ortho All flex diaphragm when used with 6% Cellulose Sulfate (CS) or with KY Jelly (placebo gel) compared with CS gel use alone over 6 months in South African women. Very few of the participants in this study had ever used diaphragms before. This combination was found to be safe with no serious adverse events or adverse events related to diaphragm use reported. Colposcopic findings were observed in 60–80% of participants. Seven severe findings were observed in those using CS in combination with the diaphragm. However, these differences were not statistically significant. The location of these findings on the external genitalia suggest that they may have been due to trauma following diaphragm insertion. The authors recommended advancing these combinations to phase III trials [20].

Buffergel in combination with a diaphragm was shown to be as effective as contraceptive as a spermicide used in combination with a diaphragm in a randomized, controlled non-inferiority trial. The point estimate pregnancy rate for Buffergel was 10.1 after 6 months of use. Buffer gel was not inferior to Gynol II, a currently available spermicide. In addition, urinary tract infection rates were lower in the Buffergel group. Side effect rates were similar in both groups, and were usually related to mild genital irritation; only 3% of participants felt that adverse events were a reason to discontinue the gel. The combination of Buffergel and diaphragm was acceptable to participants with 66% reporting that they would use this method in the future [21].

Buffergel Duet is a new device which appears to be both safe and acceptable and has potential as an over the counter product. A non-comparative study conducted in the USA among 30 couples who used the product twice per week over a period of one week, showed that 83% inserted the device correctly the first time after receiving written instructions only. The most common adverse events reported were abdominal pain. Colposcopic findings included external genital abrasions which were thought to be due to product insertion. 75% of couples were satisfied with the device with most reporting that this device was better or the same as the diaphragm [22].

### Phase III trials update, April 2006

A number of products have already advance to Phase III trials. Table 1 summarises those products, the location and progress of the Phase III trials. The first results of effectiveness trials are expected in 2007. There are a number of challenges facing the conduct of these trials including lower than expected rates of HIV incidence leading to the closure of at least one trial site. Adherence to gel was also identified as a challenge particularly in the context of no gel use, and high incidence of pregnancy among trial participants.

**Table 1:** Update of Phase IIB/III clinical trials, April 2006

Network	Products	Design	Population	Endpoints	Progress to date
Population Council	Carraguard Placebo	Ph III RCT	3 sites 6639 16–40 years	HIV	6000 accrued
HIV Prevention Trials Network (HPTN 035)	BufferGel PRO 2000 0.5% Placebo Condom only	Ph II/IIb safety and effectiveness RCT	5 sites; 800 (Ph II);	Safety; HIV	827 accrued
CONRAD	CS 6% Placebo	Ph III RCT	4 sites 2574 HR women	HIV CT, GC	738 accrued
Microbicides Development Programme (MDP)	PRO 2000 0.5% PRO 2000 2% Placebo	Ph III RCT	5 sites 9673	HIV HSV, GC	640 accrued
Family Health International (FHI)	CS 6% Placebo	Ph III RCT	2 sites 2160 HR women	HIV CT, GC	1102 accrued
Methods for Improving Reproductive Health in Africa (MIRA)	All flex diaphragm with Replens Condom only	Open-label RCT	5000	HIV HSV2 CT, GC, TV Acceptability	Completed accrual;
Family Health International (FHI)	C31G 1% Placebo	Ph III RCT	4284 HR women	HIV	Ghana closed Feb-06 2100 accrued Nigeria

Source: Trial Updates and Preliminary Data of Phase IIb/III Microbicide Trials. Guest Speaker Session. Microbicides 2006 Conference, Cape Town, South Africa, 23–26 April 2006.

CT: *Chlamydia tracomatis*

GC: *N. gonorrhoea*

TV: *Trichomonas vaginalis*

CS: Cellulose sulphate

C31G: (SAVVY)

### Future trial designs

A plenary talk by Benôit Mâsse discussed issues around future designs of microbicide trials, should an efficacious product be identified among the current generation of microbicide trials. With a number of "first generation" products in Phase III trials, consideration needs to be given to the design of future microbicide trials if one or more of these products is shown to be at least partially effective. When deciding whether to use placebo controls or active controls, it will be necessary to consider the relative effectiveness of the product, the safety profile of the two products to be compared and the populations in which these products have been tested. It may still be acceptable to use placebo controls in untested populations if there is uncertainty about any of these parameters. An alternative to superiority trials are non-inferiority trials. However, non-inferiority trials may prove more challenging, and may not even be feasible to implement because of the requirement for large sample sizes. In cases where the comparison gel is of known low effectiveness (30–40%), superiority trials may still be preferable. Add-on trials are also an option for evaluating a combination of proven products with the addition of a new product in the active arm. However, they are limited in their ability to assess monotherapy. All of these options need to be considered within the context of a strong ethical framework, where acceptability to communities is also taken into account.

## Rectal Microbicides

### Ian McGowan

Anal intercourse (AI) remains the most important risk factor for HIV-1 transmission and super infection among men having sex with men (MSM) and heterosexual anal intercourse. A growing body of epidemiological studies suggests that AI may be a relatively common practice among heterosexuals. It is clear that a safe and effective rectal microbicide will be important for both MSM and heterosexuals who practice AI.

Dr. Ian McGowan presented an overview of how rectal microbicide safety will be assessed in clinical trials. Microbicides have been evaluated in a number of animal model systems including mice and non-human primates. Epithelial damage is the main criterion for toxicity but Zeitlin et al. have documented increased vulnerability to infections such as herpes simplex 2 (HSV-2) as another index of microbicide toxicity [23]. Human intestinal explants provide a useful ex vivo/in vitro means to assess microbicide toxicity. Assessment of cytotoxicity can be performed on the basis of histology or the MTT [3-(4,5-dimethylthiazol-2-yl)-2,5-diphenyltetrazolium bromide] assay. Abner and Fletcher have both recently published detailed descriptions of this approach to toxicity assessment [24,25]. Human rectal safety studies are extremely limited but have demonstrated that nonoxynol-9 is not safe in the rectal compartment [26-28]. Epithelial disruption may be a relatively crude index of microbicide toxicity. It is conceivable that products may induce immunological changes (increased cell activation, cell recruitment, upregulation of HIV-1 co-receptors) that increase the risk of HIV transmission but are not apparent using standard histological techniques.

Dr. Peter Anton reviewed methodological challenges in designing human rectal safety studies. While the field has much to gain from experience acquired in vaginal microbicide clinical trials and design, there have been no Phase 1/Investigational New Drug (IND) rectal microbicide trials to date. The first such study is planned to start in the 3^rd ^quarter of 2006 and will evaluate the rectal safety of the vaginal formulation of UC-781. In his talk, Dr Anton reviewed many of the compartment-specific uncertainties that were addressed in the development of this protocol. These issues included regulatory challenges, the selection of safety criteria, the role of endoscopic appearance, which mucosal area should be sampled, the importance of tissue-based inflammation data, relevant behavioral data acquisition, and confounding factors that are clinically relevant such as preparatory enemas, applicator lubrication, and timing of safety and PK assessments. These discussions laid the groundwork and generated experience for the advent of rectal compartment-specific formulations to enter clinical trials testing.

Dr. Pamina Gorbach presented a comprehensive overview of the epidemiological data that is currently available on the prevalence and characteristics of anal intercourse (AI). Data sets are limited in number as well as the degree of detail that is available. Prevalence of reported AI varies across countries. The differences in prevalence of AI may be a consequence of the method used to collect data, and participants' willingness to report experience with AI. Dr. Gorbach also presented data from a number of studies in which more than half of the participants reported never using condoms during AI [29,30]. It is possible to profile heterosexual individuals that are more likely to practice AI in the US.

Dr. Alex Carballo-Dieguez reviewed the scant literature available on the topic of rectal microbicide acceptability. Acknowledging the restrictions inherent to acceptability studies in early phases of product development, he highlighted what is possible to explore concurrent with Phase 1 studies of rectal microbicides in men and women. He presented data from an ongoing volume escalation study in MSM. In this trial, MSM reported the acceptability of 5 to 50 ml of Femglide inserted rectally with or without AI. It appeared that up to 35 ml of gel was acceptable.

Jim Pickett reminded the audience that MSM and women around the world need safe, effective rectal microbicides to protect themselves from HIV and potentially other sexually transmitted infections. In 2005, microbicides, HIV/AIDS, and sexual and reproductive health advocates across the globe mobilized to form the International Rectal Microbicide Working Group (IRMWG), currently including over260 advocates, researchers and policy-makers from 29 countries linked by a global listserv and regular teleconferences featuring presentations of the latest in research. Collaborating across four continents, the group has developed a joint global advocacy strategy. He highlighted two components of IRMWG's work: 1) The collaborative process; successes and challenges of building global collaboration between advocates and researchers, and in relation to the broader microbicides movement; 2) Sharing the concrete outcomes of our advocacy and research efforts, including an analysis of current research and resource allocations in a report being released at M2006 titled"Rectal Microbicides: Investments and Advocacy" [31].

## Track C – Social and Behavioural Science

### Gita Ramjee and Neetha Morar

The social science track covered a wide range of topics including acceptability of microbicide products, ethics in research, community partnerships and involvement. In addition, clinical trial implementation issues that involved both the role of community and behavioural issues were covered in cross-track sessions.

Dr Joanne Mantell, Columbia University, New York, was invited to provide an overview of acceptability studies conducted. Since 1995, about 60 acceptability studies have been conducted, including both hypothetical studies and measuring product use and acceptability among participants within clinical trials. It must be noted however, that conducting acceptability studies among trial participants in a clinical trial setting cannot be generalised to acceptability in the general population. Furthermore, use in a clinical trials setting does not necessarily translate to preference for a product.

Empirical insights from product acceptability focused on women and their partners' acceptability of product use. This included an understanding of the prevention approach to the product. Many studies revealed that, while some women preferred covert use, majority preferred informing their male partner, especially when the woman was in a more established partnership. Use during sexual relationships was enhanced if the product increased sexual pleasure. While there were some concerns about "dry sex", reportedly practiced in many settings, research reports suggest that some wetness is welcome by both men and women [32-34].

Early lessons learned from ongoing trials suggests that adherence to product use in a clinical trial setting is based on not only disclosure to the male partner, but also on acceptability of the product by both partners. In ongoing clinical trials, there are numerous challenges in measurement of product adherence as data obtained are mainly those which are self-reported by the trial participants [35].

It is now widely believed that involving male partners may impact on product adherence. Two studies showed that men preferred to be informed of gel use by women (which maintains trust between partners) with HIV infected men being supportive of their partner's gel use and trial participation [34]. Male involvement in clinical development of Microbicides as well as product promotion campaign without determining women's autonomy is a challenge [36,37].

Additional social science issues around trial implementation were covered in sessions on community involvement and partnerships. The key issues emerging from ongoing trials was the fear of HIV testing among trial participants in communities. Participating trial sites provide extensive education to the community and have a well-defined community outreach programmes. This has proven to not only enhance community wide HIV prevention education, but assists in recruitment of trial participants [38].

Discussion on ethics of clinical research focused on the subject of violence against women. A study interviewing 24,000 women in ten countries (Bangladesh, Brazil, Ethiopia, Japan, Namibia, Peru, Samoa, Serbia and Montenegro, Thailand, and the United Republic of Tanzania) showed that 72% of women reported sexual violence, and cited their male partners as the perpetrators. This data underscored the need for expanding HIV prevention counselling to include counselling on sensitive issues such as violence, rape, etc in a microbicide clinical trial setting [39].

Standards of care in HIV Prevention clinical trials is a moving target. Increasingly, access to HIV treatment and care has become more widely available in settings where microbicide trials are conducted. Researchers and sponsors have recognised the need for referral for care for HIV positive women screened out through voluntary counselling and testing in clinical trials, and for those who become HIV positive during the course of the trial. There have been considerable advances made in addressing these issues. Many of the current trial sites have partnered with local care providers for ongoing counselling and care for women screened out of prevention clinical trials. Furthermore, partnerships have been forged with PEPFAR (President's Emergency Plan for AIDS Relief) to assist with scaling up of treatment and care. Similarly, there has been considerable advances through partnerships for facilitation of care for those women who seroconvert in prevention clinical trials.

## Track D – Community and Advocacy

### Megan Gottemoeller

2006 marked the first year in which the international Microbicides conference included a track dedicated to community and advocacy issues. This event marks a growing recognition among the scientists and research sponsors that community and civil society play a fundamental role in microbicides development, not only as trial participants or fund-raisers, but as key stakeholders who can move research implementation forward positively, productively, and in ways that meet the ultimate goal of microbicide development: to reduce people's vulnerability and risk of HIV through prevention methods that are user-friendly, and that assist people in exercising their rights to health and autonomy.

The pathway was laid out by plenary speaker Morenike Ukpong, who clarified the definitions of the numerous stakeholders who consider themselves part of the "community" involved in microbicides research. She distinguished the local, geographically or interest-defined community in which clinical trials take place from the broader community of stakeholders- better described as "civil society"- comprised of NGOs, networks, advocates, activists, and media for whom the conduct of research and development of microbicides are important, though their lives may not be directly affected by a trial. Both sets of stakeholders must be involved, though they have different needs and may be involved in different ways.

"Involvement" as defined by civil society groups includes advocating for a research agenda that reflect the priorities of various stakeholders. Microbicides 2006 demonstrated the process of dialogue between advocates and scientists on issues such as research into rectal microbicides and microbicides for use by HIV positive women. Advocates for rectal microbicides have successfully joined with scientists to identify gaps in knowledge and funding for research into microbicides that are safe and effective for use during anal sex, and have formed an internationally representative coalition to demand more research in this area [40]. HIV positive women's groups such as ICW (International Community of Women living with HIV) have been demanding research into microbicides for use by positive women for many years. At a Track D roundtable, microbicides researchers and representatives from the positive women's advocacy community were able to articulate and explore current knowledge on the scientific questions that need to be answered. Long-term safety of candidate microbicides for HIV positive women was the main concern, particularly the implications of microbicides that contain anti-retrovirals for future treatment. Without data from a proven product, it is difficult to validate indicators of long-term safety, meaning scientists can collect data but may not know how to interpret it. Discussion centered around how the field can effectively communicate that research is proceeding according to the best available knowledge and guidelines for consumer protection, while acknowledging that "long-term safety is a moving target." Although the questions may be scientific, getting the answers to the questions requires continued collaboration between researchers and the advocacy groups that represent different affected constituencies.

Also highlighted in track D was the progress of microbicides advocacy in different regions. Presentations from India [41], Nigeria [42], Canada [43], and South Africa [44] discussed the impact of advocacy and organizing, resulting in greater communication and collaboration among stakeholders in civil society and government through multi-sectoral working groups, as well as in concrete national policies such as inclusion of microbicides research in national AIDS plans. In all four countries, defining a *national agenda *for microbicides that is linked to global efforts yet reflects that country's own priorities and resources was a key factor in these advocacy successes. Presentations in Track D also highlighted the different priorities and concerns that various stakeholders have in supporting microbicides, depending on context. A mapping exercise in Southeast Asia showed that the key issues stakeholders were concerned about involved patent law and eventual access to microbicides, while women's rights groups in several countries express concern about the implications of microbicides as a technological "quick fix" that will not address underlying structural factors contributing to women's vulnerability [45]. Presenters gave examples of various "points of entry" into advocacy campaigns that respond to the contexts and concerns of these stakeholders, including framing microbicides as part of a sexual and reproductive rights agenda [44], or aligning microbicides with vaccines and treatment in demanding access to essential technologies [46].

In addition to informing advocacy strategies, research on attitudes and perceptions of sex, sexual relationships and microbicides contributes to planning for introduction, access, and marketing of proven products. Presentations and posters examining women's and men's perceptions of microbicides indicated a desire for de-linking microbicides from "infection prevention," focusing instead on hygiene, pleasure, and health in order to avoid the stigma and trust issues that have interfered with other prevention methods such as male and female condoms [47].

A crucial component of access to proven microbicides will be donor commitment, with support for both procurement and distribution strategies. A "donor panel" in the Track D programme invited representatives from donor agencies to comment on their plans and capabilities [48]. Many agencies do have mechanisms to get product to potential users; however, attention must still be paid to ensuring smooth regulatory and licensure processes, and to preparing national governments to make requests to donors. In addition, ongoing advocacy for increased funding must continue to address growing needs for the field- not only for clinical research but for numerous accompanying processes, such as developing community and civil society capacity to work with researchers, strengthening health care systems at trial sites, and ensuring sustainable care for individuals and communities involved in trials [49].

In addition to broad civil society engagement in advocacy, progress in microbicides development depends on the involvement of communities in the many sites throughout the world where clinical trials are actually taking place. There were numerous presentations and posters highlighting new models and mechanisms for engaging communities in research implementation. These included methods to support participant recruitment and retention, such as quota sampling to identify and begin conversations with at-risk women eligible for trial participation [50], and the use of GPS (Global Positioning System) technology to help research staff follow up with consenting participants enrolled in trials [51]. Additionally, presenters discussed evolving mechanisms that increase the level of participation of trial communities in planning and implementing research. Examples included using the participatory technique of cognitive mapping to engage community members in understanding and explaining community dynamics to researchers [52]; training of trial participants as peer educators [53]; and a process to increase the accountability and autonomy of a community advisory group [54].

In the six years since the first International Microbicides Conference in 2000, the recognition of the role of communities and advocates has grown exponentially. The microbicides field continues to lead other areas of health research in demonstrating how multiple stakeholders work together, learning from past lessons and creating new ways of doing things as we go. The integration of community and advocacy issues into a dedicated track within the conference is a clear demonstration of the field's commitment to this process.

## Summary

In summary, the Microbicides 2006 Conference showed the rapid advancement in the field of microbicides as a new HIV prevention technology. The conference highlighted the enormous amount of progress made in basic science with respect to product design and development. The status of the current ongoing safety and large-scale efficacy trials provided a much-needed affirmation of the progress in the field over the last couple of years. This however, does not come without challenges in future study design and implementation.

The social and behavioural track provided us with insights into social and cultural issues at community level, and the challenges in acceptability and disclosure of product use among female participants in clinical trials. The need for community involvement and partnerships was underscored by Track D participants. It was heartening to learn that a large number of microbicide advocacy groups are active in many parts of the world, not only pushing forward the microbicide agenda, but also addressing community issues and concerns in various regions.

The excellent scientific programme and participation by hundreds of delegates dedicated to microbicide research and development contributed to the resounding success of the conference in Cape Town, South Africa.

## Declaration of competing interests

The author(s) declare that they have no competing interests.
